# Surgical debulking promotes recruitment of macrophages and triggers glioblastoma phagocytosis in combination with CD47 blocking immunotherapy

**DOI:** 10.18632/oncotarget.14553

**Published:** 2017-01-06

**Authors:** Huaiyang Zhu, Lina Leiss, Ning Yang, Cecilie B. Rygh, Siddhartha S. Mitra, Samuel H. Cheshier, Irving L. Weissman, Bin Huang, Hrvoje Miletic, Rolf Bjerkvig, Per Ø. Enger, Xingang Li, Jian Wang

**Affiliations:** ^1^ Department of Biomedicine, University of Bergen, Bergen, Norway; ^2^ Department of Oncology, Shandong Chest Hospital, Jinan, China; ^3^ Department of Neurosurgery, Qilu Hospital of Shandong University, Jinan, China; ^4^ Brain Science Research Institute, Shandong University, Jinan, China; ^5^ Neuro Clinic, Haukeland University Hospital, Bergen, Norway; ^6^ Institute for Stem Cell Biology and Regenerative Medicine, Stanford University, USA; ^7^ Division of Pediatric Neurosurgery, Department of Neurosurgery, Stanford University, USA; ^8^ Department of Pathology, Haukeland University Hospital, Bergen, Norway; ^9^ Department of Neurosurgery, Haukeland University Hospital, Bergen, Norway

**Keywords:** glioblastoma, CD47, signal regulatory protein-α, phagocytosis, macrophage

## Abstract

Surgical resection is a standard component of treatment in the clinical management of patients with glioblastoma multiforme (GBM). However, experimental therapies are rarely investigated in the context of tumor debulking in preclinical models. Here, a surgical debulking GBM xenograft model was developed in nude rats, and was used in combination with CD47 blocking immunotherapy, a novel treatment strategy that triggers phagocytosis of tumor cells by macrophages in diverse cancer types including GBM. Orthotopic patient–derived xenograft tumors expressing CD47 were resected at 4 weeks after implantation and immediately thereafter treated with anti-CD47 or control antibodies injected into the cavity. Debulking prolonged survival (median survival, 68.5 *vs* 42.5 days, debulking and non-debulking survival times, respectively; *n* = 6 animals/group; *P* = 0.0005). Survival was further improved in animals that underwent combination treatment with anti-CD47 mAbs (median survival, 81.5 days *vs* 69 days, debulking + anti-CD47 *vs* debulking + control IgG, respectively; *P* = 0.0007). Immunohistochemistical staining of tumor sections revealed an increase in recruitment of cells positive for CD68, a marker for macrophages/immune cell types, to the surgical site (50% *vs* 10%, debulking *vs* non-debulking, respectively). Finally, analysis of tumor protein lysates on antibody microarrays demonstrated an increase in pro-inflammatory cytokines, such as CXCL10, and a decrease in angiogenic proteins in debulking + anti-CD47 *vs* non-debulking + IgG tumors. The results indicated that surgical resection combined with anti-CD47 blocking immunotherapy promoted an inflammatory response and prolonged survival in animals, and is therefore an attractive strategy for clinical translation.

## INTRODUCTION

Prognosis of glioblastoma multiforme (GBM) remains bleak, with a median survival of about 14.6 months and only 4% of patients surviving 5 years after the initial diagnosis [1–3]. Surgical resection is considered to be the most effective approach in the treatment of GBM patients [4–7]. Radio- and chemotherapy are also generally applied in combination with surgery to treat GBM in the clinic. Despite this multimodal treatment regimen, most GBM cases relapse within one year, and almost all relapses occur at the tumor debulking site. The failure of standardized therapies is believed to be a result of cellular and genetic inter- and intra-tumor heterogeneity, infiltration deep into the surrounding parenchyma, and a dysfunctional blood vessel supply which is unable to effectively deliver drugs to the tumor bed [8–11].

Immunotherapy is fast becoming a viable strategy for the treatment of a variety of highly aggressive human solid tumor types. The approach exploits the expression of tumor specific proteins and thus succeeds at eliminating tumor cells while sparing normal cells. A novel target for immunotherapy is CD47, a cell surface protein that is often highly expressed on human tumors and enables escape from immune surveillance. It is a cell transmembrane protein, also known as an integrin associated protein that belongs to the immunoglobulin superfamily [15]. CD47 functions as a ligand of signal regulatory protein-α (SIRPα), an inhibitory protein expressed on macrophages [16]. This interaction, also known as a “don't eat me” signal, protects cells from phagocytosis through a signaling cascade transmitted via phosphorylation of the immunoreceptor tyrosine-based inhibition motif present on the cytoplasmic tail of SIRPα [17]. Studies performed with experimental models of solid tumors including GBM as well as some hematopoietic cancers have shown that blocking the interaction between CD47 and SIRPα with anti-CD47 monoclonal antibodies (mAbs) promotes phagocytosis *in vitro* and inhibits tumor growth *in vivo* [18–23]. One of the caveats in these models, particularly in the case of GBM, is that antibody is administered to the experimental animals at an early stage in tumor development. Therefore, it remains unclear as to whether the antibody is effective on mature or recurrent tumors as is the status for GBM patients at diagnosis.

In this study, we sought to refine the strategies to evaluate the feasibility of targeting CD47 therapeutically in a clinically relevant model of GBM. We developed a protocol for surgical resection of GBM in nude rats which parallels the clinical course observed in human patients, tumor debulking followed by tumor relapse, and the model was used specifically to test combination therapy with anti-CD47 mAb. The results indicate that CD47 blocking immunotherapy might be a promising postsurgical treatment for GBM and that targeting CD47 has the potential to eliminate tumor cells driving recurrence in GBM.

## RESULTS

### Surgical debulking of GBM xenografts at 4 weeks post-implantation enhances survival

**Figure 1:**
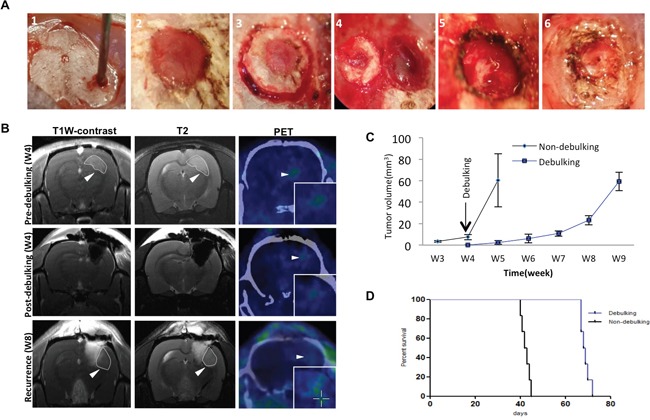
Survival in rats implanted with GBM is enhanced with surgical debulking **A**. Representative images of implantation of spheroids with a wide bore syringe (1), burr hole drilled in the skull for implantation (2), craniectomy centered around the original burr hole in preparation for tumor debulking at week 4 (3), removal of skull bone (4), removal of tumor tissue by aspiration (5), and reinsertion of resected skull bone which was fixed with cyanoacrylate glue (6). **B**. Representative images of MRI and PET-CT scans of animals at one day before debulking (week 4), one day after debulking, and tumor recurrence (week 8). Circles (MRI) and arrows (PET) highlight areas of xenograft growth. **C**. Tumor volume (mm^3^) calculated from MRI scans plotted as a function of time in weeks. Tumor was resected at week 4 after implantation. **D**. Kaplan-Meier plots illustrating survival time (implantation = day 1) of nude rats (*n* = 6/group) with or without tumor debulking (*P* = 0.0005).

Surgical debulking is a standard treatment for patients with GBM. To further understand the characteristics and cycles of GBM growth, resection and tumor recurrence in the clinic, a novel surgical debulking model using a GBM xenograft (P3) was developed. P3 spheroids (*n* = 5; 300 – 400 μm in diameter) were selected from culture and implanted in nude rats (Figure 1A). Survival time (days) was calculated on the basis that the day of implantation was day 1. MRI scans were performed to monitor tumor growth as well as debulking and recurrence (Figure 1B). At ~ 4 weeks following implantation, surgical resection was performed. PET scanning confirmed nearly complete debulking of the tumor (Figure 1B, middle panel), but also revealed that tumor cells remained within the resected margin. Thus, tumors still recurred within ~ 4 weeks of tumor debulking (Figure 1B, lower panel).

Growth curves as assessed by tumor volume on MRI between non-debulking and recurrent tumors differed (*P* = 0.001), indicating that surgery was in fact beneficial (Figure 1C). All rats survived to the end of the study with tumor debulking significantly prolonging overall survival time as assessed in survival curves (median survival, 68.5 *vs* 42.5 days, debulking and non-debulking survival times, respectively; Figure 1D).

### Vascular morphology and increased proliferation distinguish resected from non-resected xenografts

**Figure 2:**
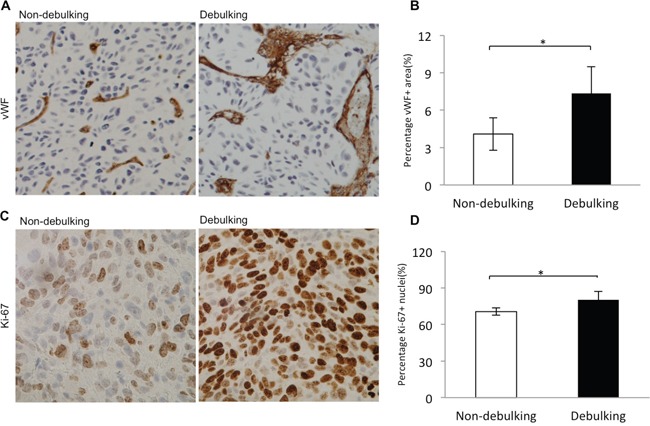
Increased proliferation index and vascular changes in debulking relative to non-debulking xenografts Immunostaining performed on sections from debulking and non-debulking tumors (5 rats per group) with the antibodies indicated. **A**. vWF (magnification 20×) and **B**. quantification of the percentage of vessel area per view (*P* = 0.001); **C**. Ki67 (magnification 40×) and **D**. quantification of percentage of Ki-67 positive cells per view (*P* = 0.028). Scale bars = 100 μm.

To address mechanisms underlying xenograft growth following surgery, vascular morphology and proliferation, hallmark pathological features of GBM, were examined in debulking and non-debulking xenografts. Morphology of the vasculature in xenografts was evaluated by immunohistochemical staining for vWf revealing vessels with a small diameter/lumen in non-debulking tumors. In contrast, vessels with larger lumen diameters were found in debulking tumors (Figure 2A). Quantification of the vessel area fraction based on vWF staining revealed that vessel area in debulking tumors was significantly greater than in non-debulking tumors (6.5% *vs* 3.5%, debulking and non-debulking tumors, respectively; *P* = 0.001; Figure 2B).

To determine whether surgery alters proliferation in tumors, immunohistochemical staining was performed for the proliferation marker Ki67. The results demonstrated that debulking tumors exhibited an increased number of proliferating tumor cells relative to non-debulking tumors (80.3% *vs* 70.6%, debulking and non-debulking tumors, respectively; Figure 2C), and the difference in the percentage of Ki67 positive cells between the two groups was statistically significant (Figure 2D, *P* = 0.028, at least 3 images were used per tumor). These results indicated that surgery influenced the vessel morphology and the proliferation index in recurrent P3 GBM xenografts.

### P3 GBM cells express CD47

**Figure 3:**
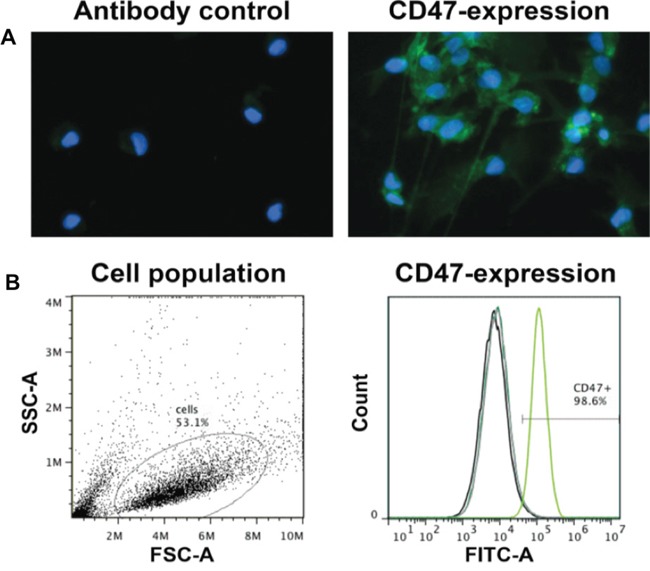
P3 GBM cells uniformly express high levels of CD47 Cell suspensions were incubated with FITC-labeled anti-CD47 antibody used for treatment in rats or IgG as a control. **A**. Fluorescent image of P3 tumor cells incubated with FITC-labeled anti-CD47 antibody (green). DAPI (blue) was used for nuclear counterstaining. **B**. Analysis of CD47 expression by flow cytometry. Forward and side scatter plots of fixed cells, and percentage of cells FITC labeled (> 98%) in the gated population.

The success of combination treatment including surgery and CD47 blocking immunotherapy is dependent on expression of CD47 in tumor cells. Human biopsy derived P3 GBM cells were therefore examined for expression of CD47 by immunocytochemistry and flow cytometry. Staining performed by immunocytochemistry demonstrated that CD47 was present on individual P3 GBM cells. The staining was specific as no positive signal was evident upon incubation of cells with only secondary antibody (Figure 3A). Analysis of CD47 expression was also performed with flow cytometry to confirm the results from immunocytochemistry and to examine the distribution of CD47 expression among P3 cells in single cell suspensions prepared from xenografts. The results confirmed that P3 GBM cells expressed CD47, and furthermore demonstrated that P3 xenograft cells uniformly expressed the protein at high levels, as more than 98% of cells were found to be positive for CD47 (Figure 3B).

### Anti-CD47 antibody promotes phagocytosis of GBM cells *in vitro*

**Figure 4:**
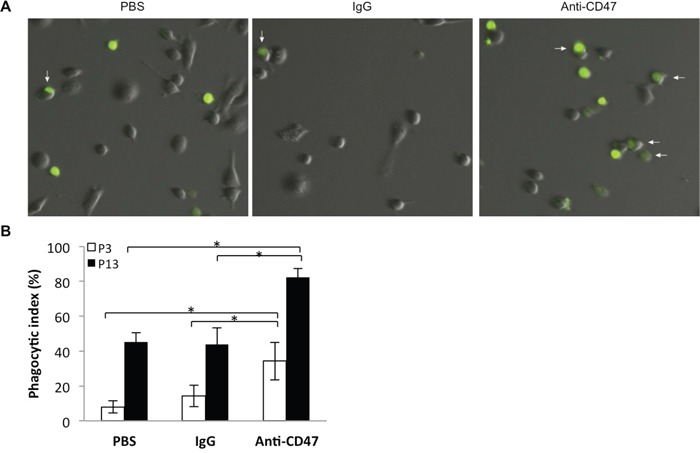
Nude rat bone marrow derived macrophages efficiently phagocytose P3 and P13 GBM tumor cells in an *in vitro* assay **A**. Representative images of derived macrophages after incubation (2 h) with CFSE (green) labeled P3 and P13 tumor cells treated with anti-CD47 antibody or controls (PBS or IgG). Co-cultures were rinsed to remove free CFSE labeled P3 cells before imaging. **B**. Bar chart displaying the phagocytic index (number of phagocytosed tumor cells per 100 macrophages) with anti-CD47 antibody and controls (IgG and PBS) for two different GBM derived CD47+ xenografts, P3 and P13. Error bars represent SD.

To observe efficiency of engulfment of GBM cells by macrophages, P3 or P13 GBM cells were labeled with CFSE and subsequently incubated with PBS, IgG control antibody, or anti-CD47 monoclonal antibody in co-cultures with macrophages differentiated from nude rat bone marrow cells. After 2 h, co-cultures were imaged under fluorescence microscopy and fluorescent macrophages were counted (Figure 4A). Phagocytic indices indicated that P3 and P13 GBM cells incubated with anti-CD47 antibody were more efficiently phagocytosed by rat macrophages than P3 and P13 cells treated with control IgG antibody or PBS (P3: 32%, 12% and 8%; and P13: 82%, 42%, and 42%; CD47, IgG and PBS control, respectively; Figure 4B). Differences in uptake were statistically significant for anti-CD47 treated P3 and P13 cells relative to the PBS and IgG antibody controls (*P* = 0.00001 and 0.00008 for P3; *P* = 0.023 and = 0.015 for P13; PBS and IgG respectively). The results however also indicated that P3 cells treated with IgG alone were more efficiently engulfed by macrophages, 12% *vs* 8%, and this result was statistically significant (*P* = 0.00962).

### Anti-CD47 antibody delays tumor growth of patient derived orthotopic GBM xenografts and prolongs the overall survival time in association with resection

**Figure 5:**
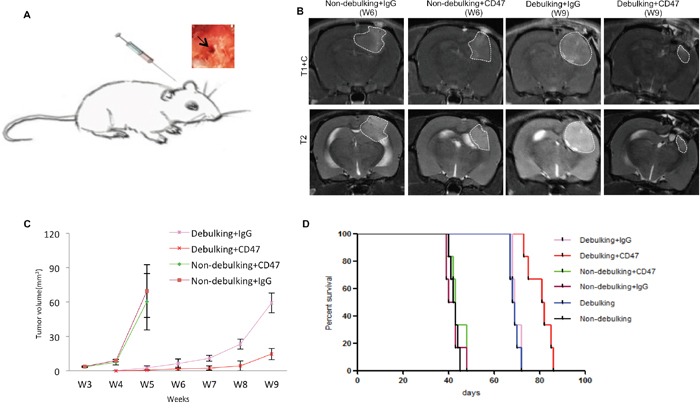
Local injection of anti-CD47 antibody inhibits tumor growth in combination with surgical debulking **A**. View of local injection of anti-CD47 antibody through the opening used to implant spheroids. **B**. Representative images of final MRI scans before sacrifice. **C**. Tumor volume derived from MRI scans and plotted as a function of time in weeks from rats treated with anti-CD47 or IgG antibodies with or without debulking. **D**. Survival of nude rats with GBM xenografts treated with anti-CD47 or IgG antibodies with or without debulking.

Efficacy of treatment in animal models is more often assessed at an early stage in the development of xenografts, namely at implantation or shortly thereafter, which differs from the time of clinical intervention in GBM patients. In order to determine whether anti-CD47 antibody was effective against tumor growth in a setting more similar to the clinic, treatment was initiated 4 weeks after implantation, a time point where tumor was clearly evident in rats by MRI (Figure 1B). Antibodies were injected directly into the tumor or resection-cavity in order to achieve a high local antibody concentration (Figure 5A), and MRI scanning was performed once a week to monitor tumor growth. Differences in tumor volumes evaluated by MRI scan were not evident in rats when treatment with anti-CD47 was initiated at 4 weeks after implantation relative to controls (Figure 5B). Tumor volume as a function of time increased similarly for treatment with anti-CD47 or control IgG antibody, as for the control group, and anti-CD47 treated animals became symptomatic within 6 weeks of implantation just as controls. Treatment with anti-CD47 antibody thus did not inhibit tumor growth when injected at 4 weeks after implantation into solid tumors.

When resection was performed in combination with initiation of anti-CD47 mAb treatment at 4 weeks, tumors still recurred in all cases (*n* = 6). The increase in tumor volume as observed on MRI scans however differed between groups (Figure 5B). Anti-CD47 mAb relative to IgG inhibited tumor growth in combination with resection, and differences in tumor volume compared to controls were statistically significant at weeks 7 (*P* = 0.0064), 8 (*P* = 0.0169) and 9 (*P* = 0.0062; Figure 5C).

Importantly, the observed delays in tumor growth in resected animals treated with anti-CD47 mAb treatment were associated with a significantly improved outcome compared to resected animals treated with IgG control antibody (81.5 days *vs* 69.0 days, anti-CD47 *vs* IgG control; *P* = 0.0007). However, survival times (~ 43 days) remained unaffected in the non-debulking group regardless of treatment with anti-CD47 or IgG antibody (*P* = 0.443; Figure 5D).

### Tumor debulking recruits M1 macrophages to the surgical site at the tumor edge

**Figure 6:**
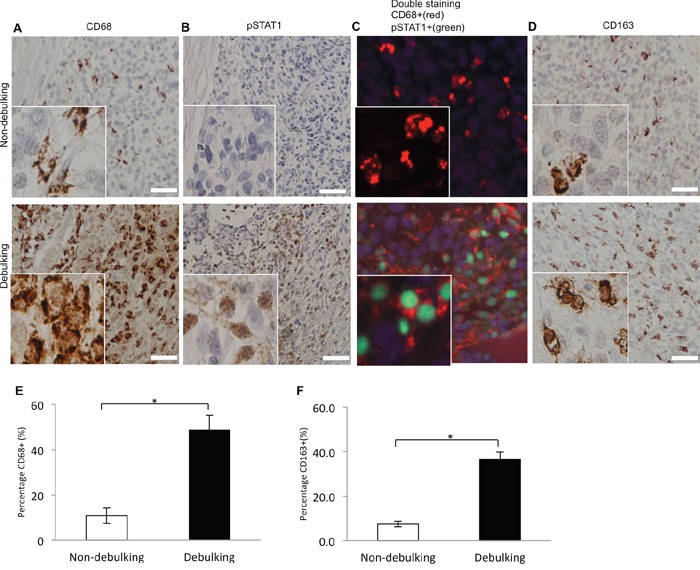
Macrophages coexpressing M1 (pSTAT1 and CD68) and M2 (CD163) polarization related markers are recruited to debulking xenografts Immunostaining performed on sections from xenografts as indicated for **A**. CD68; **B**. pSTAT1; **C**. CD68 and pSTAT1; and **D**. CD163. **E**. Quantification of macrophages based on percentage of CD68 positive cells per view (*P* = 0.021). **F**. Quantification of macrophages based on percentage of CD163 positive cells per view (*P* = 0.003). Scale bar = 100μm.

The improved efficacy of combination treatment might depend on the number and phenotype of macrophages/immune cells located within the tumor bed. To determine whether macrophages were recruited to the tumor-debulking site, staining for CD68 was first used as a general marker to identify immune cell types. Tumors from the debulking group exhibited a higher percentage of CD68+ cells (50% *vs* 10%, debulking and non-debulking, respectively). A distinct pattern of localization of macrophages was also apparent; macrophages surrounded and infiltrated the outer edge of the recurrent tumor (Figure 6A).

Macrophages have been functionally and molecularly characterized as tumor inhibiting (M1) or tumor promoting (M2). CD68 and pSTAT1 together are considered to be markers of M1 or activated macrophages while CD163 is more often associated with M2 macrophages. To determine the phenotype of macrophages in tumors, staining was performed for CD68, pSTAT1, CD68 + pSTAT1 and CD163 (Figure 6B – 6D). CD68^+^/pSTAT1^+^ double positive cells were found in the tumor debulking group whereas no pSTAT1^+^ cells were observed in the non-debulking group (Figure 6C). Tumors from the debulking group also exhibited a higher percentage of CD163^+^ cells (36.5% *vs* 7.5%, debulking and non-debulking, respectively). The localization of these M2 macrophages was similar to M1 macrophages; CD163^+^ also surrounded and infiltrated the outer edge of the recurrent tumor (Figure 6D). Quantification of the percentage of CD68+ cells (Figure 6E) and CD163+ cells (Figure 6F) per image revealed a statistically significant difference between the two groups. These results indicated that recruitment of both M1 and M2 macrophages was associated with tumor resection. Therefore, either type might promote the delay in tumor growth stimulated by tumor resection alone or in combination with anti-CD47 treatment.

### Protein expression profiles correlate with an enhanced inflammatory cytokine profile and decreased angiogenesis in debulking anti-CD47 treated xenografts

**Figure 7:**
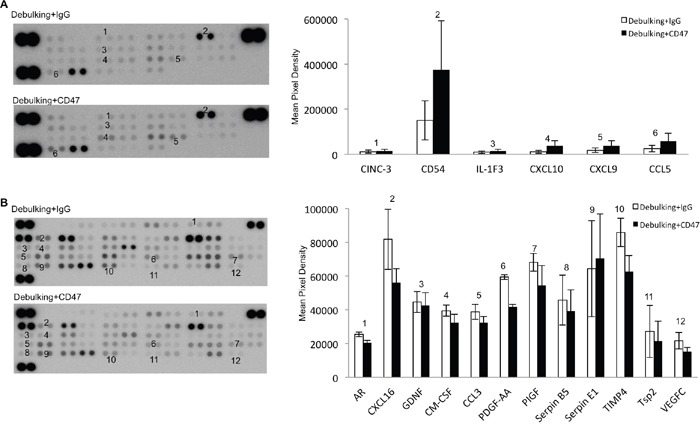
Anti-CD47 antibody treatment alters levels of cytokines and angiogenesis-associated proteins in GBM xenografts Protein arrays for **A**. cytokines and **B**. angiogenesis-associated proteins were incubated with protein lysates (400 μg) prepared from debulking tumor tissue treated with IgG or anti-CD47 as indicated. Data shown represent a 1000 s exposure to X-ray film.

To unravel the molecular mechanisms underlying the combined treatment effect, expression of cytokines and angiogenesis-associated proteins in tumor tissue from debulking+IgG and debulking+CD47 groups was evaluated on antibody protein capture microarrays. Debulking xenografts treated with CD47 antibody were characterized by significantly higher expression of cytokines/chemokines and immune cell markers CINC-3, CD54, IL-1F3, CXCL10, CXCL9, and CCL5 relative to debulking tumors treated with IgG antibody therapy (*P* < 0.05; Figure 7A). Quantification of angiogenesis associated proteins revealed that AR, CXCL16, GDNF, GM-CSF, CCL3, PDGF-AA, PIGF, Serpin B5, TIMP4 Tsp2 and VEGF-C were downregulated while Serpin E1 was upregulated in debulking tumors treated with anti-CD47 compared to debulking tumors treated with IgG (*P* < 0.05; Figure 7B). These results indicated that resection of xenografts combined with anti-CD47 treatment was associated with an enhanced inflammatory cytokine profile and a decrease in angiogenesis.

## DISCUSSION

GBM patients typically undergo surgical resection followed by chemo-/radiation therapy, but they inevitably experience relapse. At present, few animal models exist that precisely mimic this clinical course for GBM patients. Although surgical resection of GBM in mouse models has been previously reported, xenografts were initiated with glioma cell lines that do not accurately represent GBM in patients. Furthermore, the cranial window in the animal was either reconstructed with titanium mesh or not repaired [24–27]. Here, we developed a protocol for a novel xenograft surgical resection and tumor relapse model using patient derived spheroids. The model was subsequently used to examine the clinical benefits of surgery combined with a novel immunotherapy. The results demonstrated first that debulking enhanced survival in animals, and reflected the therapeutic benefit of surgical resection in patients [5, 12, 28–31]. In addition, increases in proliferation and in dilated tumor vessels (vWf) in relapsed tumor recapitulated pathological characteristics of recurrent human GBMs which display both angiogenic and invasive phenotypes [32]. Second, anti-CD47 treatment enhanced antitumor activity but only in combination with surgery. Finally, molecular expression paralleled pathological findings in tumors. These results support our model for further investigation particularly in combination therapy as well as provided potential mechanisms for the efficacy of surgery combined with anti-CD47 therapy.

A potential mechanism underlying therapeutic enhancement with anti-CD47 antibody may be the increased number/density of double positive pSTAT1-CD68 macrophages found in recurrent GBM tissue relative to non-resected xenografts. These results indicated that an increased number of macrophages, specifically activated macrophages, had been recruited to the tumor debulking site. Previous studies have demonstrated that activated macrophages in glioma tissue express high levels of CD163, CD68, CD206, and CD204 [33–36]. CD68 was used as a marker of tumor associated macrophages (TAMs) in most papers included in a meta-analysis [37] and was highly expressed on both M1 (tumor inhibiting) and M2 (tumor promoting) macrophages [38]. CD163 however was found to be expressed in M2 macrophages and has been used to distinguish between M1 and M2 cell types [39, 40]. To date, it remains difficult to differentiate between M1 and M2 TAM phenotypes in GBM. However, one study has suggested that M1 macrophages can be identified by simultaneous expression of CD68 and pSTAT1 [41]. If these markers are indeed consistent with an activated macrophage phenotype, our study is the first to illustrate their increased presence in resected GBM tumors.

The protein profiles extracted from the arrays were also consistent with more efficient recruitment of TAMs to debulking + CD47 tumors. TAMs have been shown to be recruited into tissues from the circulation by CCL5 [42, 43] which might also be expressed by macrophages [43]. In addition, activated macrophages are the cells that secrete most chemokines, including CXCL9 and CXCL10, into the tumor environment [44–47]. These cytokines were all found to be significantly upregulated in the debulking tumor + CD47 antibody group and may therefore provide a molecular explanation for the increased infiltration of macrophages into recurrent tumor as well as antibody stimulated antitumor activity in these macrophages. CINC-3 is a strong chemotactic factor for neutrophils and was found to be downregulated in the debulking tumor + CD47 antibody group. The biological effect of decreased CINC-3 on tumor growth is unclear and should be investigated in future studies. CD54 (ICAM-1) is a member of the immunoglobulin superfamily and is expressed on endothelial cells, epithelial cells, and fibroblasts, as well as T-cells, B-cells, dendritic cells, macrophages, and eosinophils [48]. Studies have demonstrated that CD54 plays an important role in facilitating tumor invasion and metastases [49, 50]. In our study, CD54 was found to be significantly upregulated in the debulking tumor + CD47 antibody group. Increased expression may be due to an increase in the number of macrophages recruited to recurrent tumor. The result was consistent with a study showing that CD47 blockade leads to increased macrophage infiltration in tumors [51]. In addition, angiogenesis-promoting proteins, such as AR, CXCL16, GDNF, GM-CSF, CCL3, PDGF-AA, PIGF, are downregulated. These results provide a molecular basis for why combination therapy led to significantly slower growth than in controls. Thus, all together, these results provide a potential mechanism for the enhanced anti-tumor activity observed with blockade of CD47-SIRPa in the context of surgical debulking.

Our observation that anti-CD47 therapy had no effect on the primary tumor mass in nude rats in the absence of debulking, corroborates previous results. In a previous study, anti-CD47 antibody elicited a profound effect in the treatment of solid tumors including GBM; however, efficacy of anti-CD47 blockade was highly correlated with tumor size [23]. This result was surprising as our *in vitro* experiments had shown significant phagocytosis of tumor cells in the anti-CD47 group, but it was only smaller size after debulking in rats in the anti-CD47 group that was associated with significantly longer survival compared to controls. The results indicate that anti-CD47 therapy effectively enhanced treatment of recurrent tumors in the context of postsurgical tumor resection and thus might potentially improve overall survival of patients with GBM.

One of the potential drawbacks of anti-CD47 therapy is the method of delivery. Rats had also been treated with anti-CD47 antibody by tail vein injection, but no efficacy was observed (data not shown). The reasons for failure of the treatment administered by this method may be the inability of antibody to cross the blood–brain tumor barrier and/or an insufficient antibody concentration for the inhibition of tumor growth in the rat GBM model. Other treatment options such as temozolomide, the angiogenesis inhibitor endostatin or encapsulated therapeutic stem cells have exhibited efficacy when locally administered in animal models [25, 26, 52]. Therefore, anti-CD47 therapy was administered by local injection immediately following debulking of tumors. Local delivery or alternative delivery strategies should, thus, be considered over systemic delivery methods when administering antibody therapy in clinical trials with brain tumors.

In conclusion, CD47 was found to be highly and uniformly expressed on P3 patient derived GBM cells. Inhibiting CD47 with anti-CD47 mAbs enabled phagocytosis of GBM cells by macrophages *in vitro*, and postsurgical treatment by local injection of anti-CD47 serves as an effective method *in vivo*. These results support the rationale for evaluating clinical efficacy of anti-CD47 therapy post-surgically in human patients with GBM. In addition, the statistically significant enhancement of survival in combination treatment of recurrent tumor is possibly driven by recruitment of CD68+/pSTAT1+ macrophages and inhibition of angiogenesis. Additional studies are necessary to address the relationship between efficacy of anti-CD47 treatment and the density of TAMs in human GBMs, and the feasibility of combining CD47 blocking immunotherapy with cytokines that modulate macrophage recruitment and activation to treat GBM in patients.

## MATERIALS AND METHODS

### Ethics statement

The collection of human biopsy tissue for research purposes was approved by the Regional Ethical Committee (REK Vest, project number 013.09; Bergen, Norway), and written informed consent was obtained from patients before surgery. All procedures and experiments involving animals in this study were approved by the National Animal Research Authority (Norway) and conducted according to the European Convention for the Protection of Vertebrates Used for Scientific Purposes.

### Preparation of patient GBM-derived spheroids

Patient GBM-derived spheroids (P3, P13) were originally generated *in vitro* in our laboratory from primary tumor tissue as previously described [32]. Spheroids were subsequently implanted into nude rat brains in order to establish clinically and biologically relevant GBM xenografts. Xenografts have been maintained and expanded through serial passage in animals with the generation of spheres *in vitro* before the subsequent implantation *in vivo*. Implantations for tumor debulking experiments were standardized through the selection of a pool of *in vitro* generated spheres (*n* = 5; 300 – 400 μm in diameter) which developed into phenotypically identical xenografts (angiogenic and invasive) within the expected timeframe of ~ 30 days in nude rats.

### Spheroid implantation

Immunocompromised athymic nude rats (rnu -/−) were used for all implantations. These animals have been previously shown to be a favorable host for serial passage of human GBM xenografts, and their size makes them amenable to experimental procedures including surgeries and MRI and PET scans. For implantations, rats were anesthetized (3.5 mL/kg) with a mixture of Fentanyl (10 mL at 50 mg/mL; Hameln Pharma; Halmeln; Germany) and Domitor (1 mL at 1 mg/mL; Orion Pharma; Espoo, Finland), and fixed in a stereotactic frame (Benchmark; Neurolab, St Louis, MO). A small hole was made in the skull with a bit (2.9 mm in diameter), and human GBM spheroids (*n* = 5) were slowly injected through a wide bore Hamilton syringe (Hamilton; Reno, NV, USA) into the brain of athymic nude rats (*n* = 40; Figure 3A) at the following coordinates: 1 mm posterior to the bregma and 3 mm to the right of the midline suture at a depth of 2.5 mm. The incision was closed with 4/0 interrupted simple sutures and cleaned with alcohol.

### MRI and PET-CT scanning

MRI was performed on a 7T Pharmascan (Bruker; Ettlingen, Germany). Sequence details have been described previously [53]. Brain MRI was performed to evaluate tumor growth with pre-/post-contrast T1-weighted sequences. PET-CT scans were performed using a nanoScan PC PET/CT (Mediso Medical Imaging Systems Ltd, Budapest, Hungary) with F18-FET performed through tail vein injection with a dose of 15 MBq per rat.

### Surgical tumor debulking

Tumor growth in nude rats was monitored by MRI scan, and determined to be sufficient at ~ 4 weeks after implantation for debulking and antibody experiments. Animals were anesthetized and fixed in the stereotactic frame as for implantations, and respiration was closely monitored. The previous incision was extended, and surgical retractors were used to open the surgical field. The previous burr hole used for spheroid implantation was identified. A craniotomy (5 mm in diameter) was centered on the previously made burr-hole using a high-speed dental drill (diameter of 0.5 mm) under a dissecting microscope. Tumor was exposed with a durotomy and gently dissected as much as possible along the interface between tumor and normal brain tissue with a dissecting microscope or an aspirator. Hemostasis was achieved with a hand-held electrocautery pen with a fine needle tip, and the surgical cavity was rinsed with sterile saline. The resected skull bone was reinserted after the resection and fixed with cyanoacrylate glue. The incision was closed with 4/0 interrupted simple sutures and cleaned with alcohol; antibiotics however were not administered. Resected animals underwent immediate post-operative MRI scanning and were returned to their cages when fully recovered. Animals were closely monitored thereafter for neurological deficits such as seizures, paresis, and significant weight loss.

### Histopathology, immunohistochemistry and quantitative analysis

Brains were fixed in 4% formalin, embedded in paraffin, and sectioned (5 μm). Sections were stained with hematoxylin and eosin, and examined by a neuropathologist under a light microscope. For immunohistochemical analysis, sections were deparaffinized and rehydrated, and pretreated for 25 min in citrate buffer (pH 6.0) at 100°C followed by cooling for 50 min at 4°C in a refrigerator. Endogenous peroxidase was quenched with a 5 min incubation in Peroxidase Blocking Solution (K4007; DAKO; Glostrup; Denmark). Sections were blocked with DAKO Protein Block (X0909; DAKO) for 30 min and incubated overnight at 4°C with primary antibody. The antibodies used were the following: Ki67 (1:75, MIB-1 clone, DAKO); von Willebrand Factor (1:500, DAKO); CD68 (1:100, ab31630, Abcam; Cambridge, MA, USA); p-STAT1 (1:300, Tyr701, Santa Cruz Biotechnology; Dallas, TX, USA); and CD163 (1:100, Serotec; Oxford, UK). Detection was performed using biotinylated secondary antibody conjugated to peroxidase (40 min incubation; anti-mouse, K4007; anti-rabbit, K4011; DAKO) and DAB+ as the substrate (3 min; K4007; DAKO) according to the manufacturer's instructions. Sections were counterstained with hematoxylin, dehydrated and mounted in Entellan (Merck; Darmstadt, Germany). Histological analysis was performed using a Nikon Eclipse E600 light microscope (Nikon; Tokyo, Japan). Quantification of Ki67, von Willebrand Factor (vWF), CD68 and pSTAT1 (5 tumors per experimental group, 3 different areas per tumor) was performed with the imaging software NIS-Elements BR 4.11.00 64-bit (Nikon). The number of Ki67 and vWF positively stained cells were counted in 9 non-overlapping randomly selected areas (at 400× magnification) from at least three different tumor-bearing brains from each group.

### Single cell suspensions

Single cell suspensions were prepared by incubating P3 and P13 xenograft material with trypsin-EDTA (Lonza Ltd.; Basel, Switzerland) for 10 min in a shaking water bath at 37°C, followed by gentle trituration with a 1000 μl pipette tip. Cells were subsequently used for immunocytochemistry and flow cytometry or labeled with 5 μM carboxyfluorescein succinimidyl ester (CFSE) for *in vitro* phagocytosis assays.

### Immunocytochemistry (ICC) and flow cytometry

For ICC, single cells were seeded onto coverslips in a 24-well plate, allowed to attach overnight, rinsed twice with phosphate-buffered saline (PBS; Dulbecco's phosphate-buffered saline; Sigma-Aldrich; St. Louis, MO, USA), fixed for 10 min in 4% paraformaldehyde (PFA, Thermo Scientific, Rockford, USA), and rinsed three times with PBS. Coverslips were blocked in 0.5 % bovine serum albumin blocking buffer for 15 min, incubated with primary anti-CD47 antibody (BioXCell; Beverly, MA, USA) for 45 min at 37°C, rinsed twice with PBS, incubated with FITC-conjugated secondary anti-mouse antibody (1:200; Southern Biotech; Birmingham, Alabama, USA) for 45 min at 37°C, rinsed twice in PBS, and mounted in Vectashield mounting medium (Vector Laboratories; Burlingame, California, USA) with DAPI. Coverslips were imaged with fluorescence microscopy (Nikon TE2000 microscope; Nikon; Tokyo, Japan). Cells stained with secondary antibody only were used as the control. For flow cytometry, single cell suspensions were immediately fixed in 4% PFA for 10 min followed by a wash with PBS, 15 min blocking in 0.5% BSA, and incubation with anti-CD47 or isotype control antibody for 20 min at room temperature. CD47 staining was acquired on an AccuriC6 instrument (Becton Dickinson; Erembodegem, Belguim), and analyzed in FlowJo (TreeStar Inc.; Ashland, Oregon, USA). Positive gates were set on the basis of the isotype control staining.

### *In vitro* phagocytosis assay

Macrophages for *in vitro* experiments were differentiated from bone marrow cells harvested from femurs of athymic nude rats (HsdHan™: rnu/rnu Rowett). Femurs were collected from rats sacrificed by CO_2_ inhalation and dislocation of the neck, and attached tissue was removed. Both ends of each femur were cut with sterile scissors, and the bone marrow was flushed out into a 70 μm cell strainer in a petri dish with sterile PBS expelled from a syringe with a 22-gauge needle. Red blood cells were lysed with Red Blood Cell Lysing buffer (Sigma-Aldrich) according to the manufacturer's protocol. Bone marrow cells were suspended in Dulbecco's Modified Eagle Medium (DMEM, Sigma-Aldrich) supplemented with 10 ng/mL granulocyte-macrophage colony-stimulating factor (GM-CSF; Peprotech; Rocky Hill, NJ, USA), 10% fetal bovine serum, non-essential amino acids, 100 U/mL Pen/Strep and 400 μM L-glutamine (Cambrex; East Rutherford, NJ, USA), and cultured for 6 days in non-tissue culture treated 10 cm dishes (BD Biosciences; Franklin Lakes, NJ, USA). Differentiated macrophages were detached in 10 mM ethylenediaminetetraacetic acid (EDTA; Merck) using a cell scraper and viable macrophages (50,000/well) were seeded into the wells of a 24-well plate. After culture overnight, complete DMEM was replaced with serum-free DMEM 2 h before introducing CFSE labeled P3 or P13 GBM cells (*n* = 200,000) to the macrophages (*n* = 50,000). This ratio of tumor cells to macrophages *in vitro* roughly mimics the situation *in vivo*, i.e. number of tumor cells is > the number of macrophages. After a 2 h incubation at 37°C, the anti-human CD47 or IgG1 antibody (10 μg/mL) and PBS as control were added to the co-cultures, and imaging was performed after rinsing wells four times with PBS (1 mL) to remove non-phagocytosed P3 and P13 GBM cells. All co-culture experiments were performed three times with at least three parallels, and random images were taken from each of the parallels for each condition. The phagocytic index was calculated by counting the number of phagocytosed tumor cells per 100 macrophages in 3×3 images for each condition.

### Antibody treatment

Four weeks after spheroid implantation, the animals were grouped into the following six different experimental groups: tumor non-debulking (*n* = 6); tumor non-debulking with IgG isotype control treatment (MOPC-21; BioXCell; *n* = 6); tumor non-debulking with anti-human CD47 antibody treatment (B6.H12; BioXCell; *n* = 6); tumor debulking (*n* = 6); tumor debulking combined with IgG isotype control treatment (*n* = 6); and tumor debulking combined with anti-human CD47 antibody treatment (*n* = 6). The two antibodies were resolubilized in 1×PBS at a concentration of 5 mg/mL, and 50 μL were administered once every two days by local injection through the implantation burr hole starting from 4 weeks post-implantation and continuing until the experiment was terminated. Rats underwent MRI imaging (w/wo contrast) weekly to monitor tumor growth. Rats were sacrificed when neurological deficits such as seizures, paresis, and significant weight loss had occurred. Tumors were removed and either formalin fixed for histology or snap frozen in liquid N_2_ for protein extraction.

### Cytokine and human angiogenesis antibody protein capture microarrays

Protein lysates were prepared from snap frozen xenografts, and proteins (400 μg) were incubated with the Rat Cytokine Array Kit, Panel A, and the Human Angiogenesis Array Kit (R&D Systems; Minneapolis, MN, USA) according to the manufacturer's instructions. Collection and quantification of pixel densities from membranes was performed using the Fuji Las3000 (Fujifilm; Tokyo, Japan) and Image Reader LAS-3000 (Version 2.2; Fujifilm). Arrays for all samples were performed in duplicate, and an average density was calculated for each corresponding protein probed on the arrays based on the two pixel densities determined for each experiment.

### Statistical analysis

Brain MRI was performed to evaluate tumor growth and the tumor volume was evaluated with OsiriX 5.8.2. Survival was analyzed by a log-rank test based on the Kaplan-Meier test using Graphpad Prism software. Differences between pairs of groups were determined by the unpaired 2-tailed Student's t-test. *P-*values < 0.05 were considered significant.
